# In silico based analysis to explore genetic linkage between atherosclerosis and its potential risk factors

**DOI:** 10.1016/j.bbrep.2023.101574

**Published:** 2023-11-07

**Authors:** Hossain Mohammad Hridoy, Md. Nasim Haidar, Chadni Khatun, Arnob Sarker, Md. Pervez Hossain, Md. Abdul Aziz, Md. Tofazzal Hossain

**Affiliations:** a Bioinformatics and Structural Biology Lab, Department of Biochemistry and Molecular Biology, University of Rajshahi, Rajshahi, Bangladesh; b Department of Biochemistry and Molecular Biology, University of Rajshahi, Rajshahi, Bangladesh; c Department of Electrical and Electronic Engineering, Rangpur Engineering College, Rangpur, Bangladesh

**Keywords:** Atherosclerosis, Hub proteins, Genetic linkage, Risk factors, Biomarkers, Cardiovascular disease

## Abstract

Atherosclerosis (ATH) is a chronic cardiovascular disease characterized by plaque formation in arteries, and it is a major cause of illness and death. Although therapeutic advances have significantly improved the prognosis of ATH, missing therapeutic targets pose a significant residual threat. This research used a systems biology approach to identify the molecular biomarkers involved in the onset and progression of ATH, analysing microarray gene expression datasets from ATH and tissues impacted by risk factors such as high cholesterol, adipose tissue, smoking, obesity, sedentary lifestyle, stress, alcohol consumption, hypertension, hyperlipidaemia, high fat, diabetes to find the differentially expressed genes (DEGs). Bioinformatic analyses of Protein-Protein Interaction (PPI), Gene Ontology (GO), and Kyoto Encyclopedia of Genes and Genomes (KEGG) were conducted on differentially expressed genes, revealing metabolic and signaling pathways (the chemokine signaling pathway, cytokine-cytokine receptor interaction, the cytosolic DNA-sensing pathway, the peroxisome proliferator-activated receptors signaling pathway, and the nuclear factor-kappa B signaling pathway), ten hubs proteins (CCL5, CCR1, TLR1, CCR2, FCGR2A, IL1B, CD163, AIF1, CXCL-1 and TNF), five transcription factors (YY1, FOXL1, FOXC1, SRF, and GATA2), and five miRNAs (mir-27a-3p, mir-124–3p, mir-16–5p, mir-129-2-3p, mir-1-3p). These findings identify potential biomarkers that may increase knowledge of the mechanisms underlying ATH and their connection to risk factors, aiding in the development of new therapies.

## Highlights

• Comprehensive transcriptomics analyses show genetic association of atherosclerosis with various risk factors.
• Candidate biomarkers for atherosclerosis include RNA (transcripts, miRNAs) and hub proteins, key signaling and regulatory molecules.
• Possible molecular pathways involving atherosclerosis.
• New gene-based recommendations for disease diagnosis.
• Molecular signatures of biomarkers for treatment of atherosclerosis.

## 1 Introduction

In the world, cardiovascular diseases (CVD) continue to be the primary reason for mortality, making up approximately 18 million fatalities each year, despite significant therapeutic advances in recent years. These figures are expected to rise to 24 million global deaths per year by 2030, with a daily average of more than 66,000, and an overall cost of more than one trillion USD [1,2]. The primary underlying factor is atherosclerosis (ATH), which ranks as the primary worldwide contributor to cardiovascular disease fatalities [3]. ATH is defined by a progressive disease caused by the build-up of plaque in arteries [4], that is prone to fatal clinical outcomes like acute myocardial infarction and sudden cardiac arrest. Even though ATH develops naturally in individuals, early lesions in large and medium-sized arteries primarily turned into advanced plaques, and it is this latter group of plaques that are responsible for the bulk of acute ischemic cardiovascular events. It is a long-term, intricate pathological condition affecting immunology, metabolism, inflammation, and oxidative stress [5,6].

Atherosclerosis advances primarily as a result of lipids, especially cholesterol-laden low-density lipoprotein (LDL) and other lipoprotein particles that contain apolipoprotein B (apoB), such as very low-density lipoprotein(a) [Lp(a)] [7,8]. The etiology of ATH has been associated to conventional risk factors like smoking, obesity, diabetes, hypertension, hyperlipidemia, homocysteinemia, hypercholesterolemia, immune damage, and genetic factors. However apart from these risk factors, infection-related inflammation as well as endothelial dysfunction is being investigated as a possible cause of the disease. Because of the disease's complex etiology and multiple comorbidities, identifying ATH biomarkers is critical for improving patient care and lowering disease risk [[9], [10], [11], [12], [13]].

**Fig. 1:**
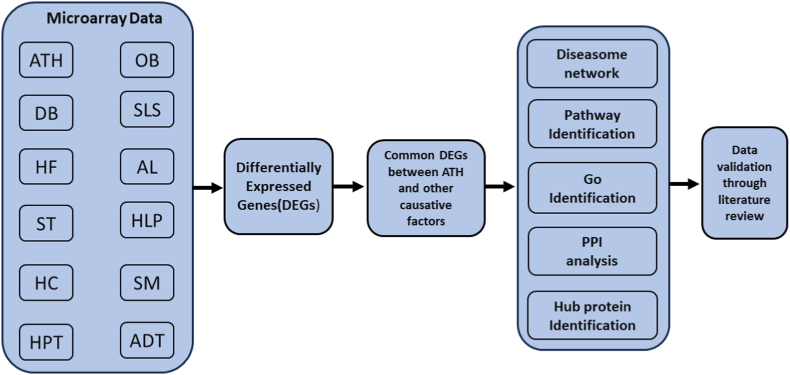
A diagrammatic presentation of the network based methodology used in this research, where ATH = Atherosclerosis, OB = Obesity, DB = Diabetes, SLS = Sedentary Lifestyle, HF = High Fat, AL = Alcohol, ST = Stress, HLP = Hyperlipidemia, HC = High Cholesterol, SM = Smoking, HPT = Hypertension, ADT = Adipose Tissue.

In gene networks, a hub gene refers to a cluster of interlinked genes that typically holds important role in gene control and biological functions. Understanding the fundamental biological mechanisms opens up new opportunities for identifying biomarkers and exploring potential drugs [14,15]. Advancements in biotechnology have made high-throughput data, including genomic, proteomic, and metabolomics data, more accessible. This type of data supports comprehensive scientific research and can aid in early diagnosis, predicting prognosis, and investigating molecular mechanisms for various diseases [16]. Consequently, in our research, we embraced a systems biology-oriented strategy to identify specific molecular biomarker transcripts (i.e., mRNAs), hub proteins, and pathways linked to atherosclerosis in relation to ATH-associated risk factors (see Fig. 1).

To achieve this, we utilized the GSE100927 dataset, comprising 69 atherosclerotic carotid artery groups and 35 control carotid artery groups. Differentially expressed genes (DEGs) were classified into upregulated and downregulated categories through GEO2R analysis of these samples. Subsequently, these widely observed DEGs were examined within human biomolecular networks like protein-protein interaction (PPI) networks to identify pivotal signaling molecules (hub proteins) and molecular pathways. This approach led to the identification of candidate genes that may influence the effects of ATH risk factors, which were then corroborated through a literature review.

The microarray gene expression datasets of ATH (GSE100927), OB (GSE60403), DB (GSE25724), SLS (GSE1786), HF (GSE68231), AL (GSE20489), ST (GSE13712), HLP (GSE1010), HC (GSE6054), SM (GSE6264), HPT (GSE703), ADT (GSE18612) were acquired from NCBI-GEO.

## 2 Materials and methods

### 2.1 Microarray gene expression datasets

We analysed microarray datasets for gene expression to investigate the molecular connections of different factors with atherosclerosis (ATH). All the datasets utilized in this research were sourced from the National Center for Biotechnology Information's (NCBI) Gene Expression Omnibus [17], with Affymetrix Human DNA arrays being the default choice unless otherwise specified. The following gene expression datasets were examined in this study: GSE100927, GSE60403, GSE25724, GSE1786, GSE68231, GSE20489, GSE13712, GSE1010, GSE605, GSE6264, GSE703, and GSE18612. The ATH dataset (GSE100927) was taken from gene expression profiling of human atherosclerotic arteries [18]. The obesity (OB) dataset (GSE60403) was acquired from gene expression arrays of cord blood from obese pregnant women [19]. The diabetes (DB) dataset (GSE25724) was obtained from microarray analysis of human islets of type 2 diabetics patients [20]. The sedentary lifestyle (SLS) dataset (GSE1786) was taken from the gene expression microarray analysis of healthy sedentary men characterized by no participation in regular exercise for more than once weekly [21]. The dataset labelled as high-fat diet (HF) (GSE68231) comprises Affymetrix Human Genome data derived from the vastus lateralis (VL) muscle of 50 subjects in each group, selected both before and after a three-day period of following a high-fat diet [22]. The alcohol (AL) dataset (GSE20489) was taken from the gene expression microarray analysis of blood samples during acute ethanol exposure [23]. The stress (ST) dataset (GSE13712) was derived from gene expression profiles of youthful and aged endothelial cells subjected to static and laminar shear stress conditions [24]. The hyperlipidemia (HLP) dataset (GSE1010) originated from gene expression arrays of blood-derived cell lines from individuals with familial combined hyperlipidemia [25]. The hypercholesterolemia (HC) dataset (GSE6054) was generated by analysing gene expression data from monocytes of patients diagnosed with familial hypercholesterolemia [26]. The smoking (SM) dataset (GSE6264) was compiled from gene expression profiles of lymphoblast cell lines obtained from both smokers and non-smokers [27]. The hypertension (HPT) dataset (GSE703) was derived from microarray analysis of gene expression in peripheral blood cells [28]. The adipose tissue (ADT) dataset (GSE18612) was taken from gene expression profiles of epicardial adipose tissue [29].

### 2.2 DEG identification

We employed transcriptomics datasets to perform a differential gene expression analysis for ATH in the presence of eleven risk factors. To ensure comparability across different platforms and experimental setups, we initially standardized the gene expression data for both disease and control states using the Z-score (or zero mean) normalization method [30]. This normalization method utilized the mean and standard deviation for each sample in the gene expression matrix. The expression value of gene i in sample j, denoted as gij, was transformed into Zij through a calculation process.

$$Z_{ij}=\frac{g_{ij}-\text{mean}\left(g_{i}\right)}{\text{SD}\left(g_{i}\right)}$$

here, SD represents the standard deviation. This conversion facilitates the comparison of gene expression levels among different samples and diseases. The gene expression datasets underwent normalization through log_2_ transformation, and the unpaired student t-test was applied. Ultimately, genes with p-values below 0.05 and an absolute log fold change (log FC) exceeding 1.0 were selected as statistically significant differentially expressed genes (DEGs).

### 2.3 Identifying Gene Ontology and pathways through gene set enrichment analysis (GSEA)

To assess the biological relevance of the identified DEGs, we conducted gene set enrichment analysis and pathway analysis using EnrichR. This allowed us to pinpoint the significant Gene Ontology terms and KEGG pathways that were enriched with DEGs [31,32]. We considered enrichment results as statistically significant if the p-value was less than 0.05.

### 2.4 Discovering regulators of DEGs at the transcriptional and post-transcriptional levels

We employed TF-target gene interactions from the JASPAR database to identify transcription factors (TFs) based on their network characteristics. This enabled us to identify TFs that regulate DEGs at the transcriptional level [33]. Furthermore, we used topological parameters to discover regulatory miRNAs that influence DEGs at the post-transcriptional level, utilizing miRNA-target gene interactions from TarBase and miRTarBase [[34], [35], [36]].

### 2.5 Analysing PPI to find hub proteins

We utilized the STRING protein interactome database to build a protein-protein interaction (PPI) network entered on the proteins encoded by the DEGs [37]. For visual analysis of the PPI network, we employed Cytoscape (v3.9.1) [38,39]. The PPI network was depicted as an undirected graph, with nodes representing proteins and edges denoting protein interactions. To identify highly interconnected proteins, known as hub proteins, within the network, we used the Cyto-Hubba plugin in Cytoscape [40,41], with degree metrics serving as the basis for assessment [42,43].

## 3 Result

### 3.1 Detecting differentially expressed genes via the analysis of microarray gene expression datasets

**Fig. 2:**
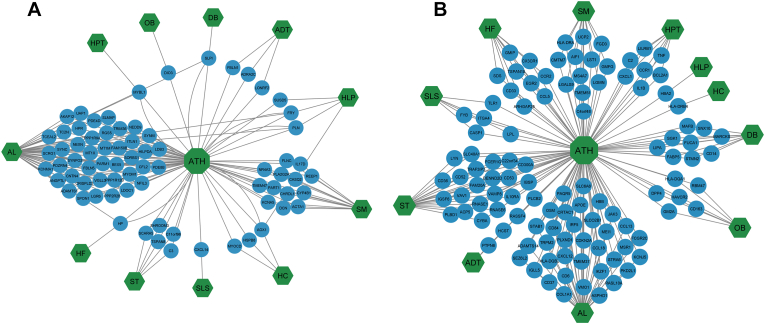
Network for up-regulated (A) and down-upregulated (B) gene of atherosclerosis (ATH) with high cholesterol (HC), adipose tissue (ADT), smoking (SM), obesity (OB), sedentary life style (SLS), stress (ST), Alcohol consumption (AL), Hypertension (HPT), Hyperlipidemia (HLP), High Fat (HF), Diabetes (DB). The target is presented in the centre by octagon-shaped node (green color) and the eleven hexagon-shaped nodes (green color) represent the risk factors. The other circle-shaped blue-colored nodes are common genes between ATH and its risk factors. (For interpretation of the references to color in this figure legend, the reader is referred to the Web version of this article.)

We examined the gene expression dataset related to ATH and identified a total of 639 differentially expressed genes (DEGs) in ATH patients compared to control samples. Among these, 167 genes exhibited up-regulation, while 472 genes displayed down-regulation. We conducted several stages of statistical analysis on the mRNA microarray data associated with the eleven risk factors to explore the relationship between ATH and these risk factors. Thus, we choose the most important Up and Down-regulated genes for each risk factor. We found 243, 1360, 1788, 378, 504, 17670, 2516, 243, 396, 200, 1182, 639 DEGs from SM, OB, DB, SLS, HF, AL, ST, HLP, ADT, HPT, HC, ATH datasets respectively. We then determined common DEGs between the ATH and the earlier mentioned factors. The ATH shared significant DEGs with SM, OB, DB, SLS, HF, AL, ST, HLP, ADT, HPT, HC, and ATH in the following numbers: 34, 16, 10, 7, 11, 82, 37, 5, 7, 9, and 7. In order to uncover statistically significant connections between these risk factors and ATH, we built up diseasome association networks centred around ATH to identify significant associations among these risk factors (Fig. 2 and Table 1).

**Table 1:** Gene Ontology concepts and KEGG pathways that are important for understanding ATH and risk factors such as SM, OB, DB, SLS, HF, AL, ST, HLP, ADT, HPT, HC, ATH.

Biological Process (BP)			
GO ID	Term/pathway	Genes	Risk factors
GO:0032387	negative regulation of intracellular transport	CD84, PLN, LGALS9, CD300A	AL, HLP, SM, ST
GO:0043301	negative regulation of leukocyte degranulation	CD84, CCR2, LGALS9, CD300A	AL, HF, OB, SM, ST
GO:0001819	positive regulation of cytokine production	PDE4D, TNF, HAVCR2, CYBA, HILPDA, IRF5, CD14	AL, HPT, OB, ST
GO:0032757	positive regulation of interleukin-8 production	CD14, IL1B, TLR1, LGALS9, TNF, IL17D	DB, HPT, SLS, SM
GO:2000484	positive regulation of interleukin-8 secretion	CD14, TLR1, LGALS9	DB, SLS, SM, ST

Cellular Component (CC)			
GO ID	Term/pathway	Genes	Risk factors
GO:0045334	clathrin-coated endocytic vesicle	APOE, HLA-DQB2, HLA-DRB4, HLA-DQA1	AL, HC, OB
GO:0030669	clathrin-coated endocytic vesicle membrane	APOE, HLA-DQB2, HLA-DRB4, HLA-DQA1	AL, HC, OB
GO:0005887	integral component of plasma membrane	CD84, PLXND1, SORBS1, TRPM2, SLC6A6, CD6, SLCO2B1, SLMAP, STAB1, CD37, LGR6, STRA6, TSPAN10, CD33, CCR2, MSR1, CD52, IGSF6, FCER1G, TSPAN8, SCARA5, SLC40A1, CD36	AL, HF, ST
GO:0071556	integral component of luminal side of endoplasmic reticulum membrane	HLA-DRB4, HLA-DQA1, HLA-DRA	HC, OB, SM
GO:0005764	lysosome	FUCA1, LIPA, DPP4, GM2A, HLA-DQA1, HLA-DRA, DCN, LGMN	DB, OB, SM

Molecular Function (MF)			
GO ID	Term/pathway	Genes	Risk factors
GO:0019956	chemokine binding	CX3CR1, CCR1, ITGA4	HF, HPT, SLS
GO:0004950	chemokine receptor activity	CX3CR1, CCR1, CCR2	HF, HPT, OB
GO:0032395	MHC class II receptor activity	HLA-DQB2, HLA-DQA1, HLA-DRA	AL, OB, SM
			
GO:0004435	phosphatidylinositol phospholipase C activity	PLCB2, CCL5, CCR1	ADT, HF, HPT
GO:0004629	phospholipase C activity	PLCB2, CCL5, CCR1	ADT, HF, HPT

KEGG pathways			
KEGG ID	Pathway	Gene in pathway	Risk factors
hsa04062	chemokine signaling pathway	CCL13, CXCL12, CCL18, JAK3, CX3CR1, CCL5, CCR2, CCR1, CXCL1, CXCL14, LYN, VAV1	AL, HF, HPT, OB
hsa04060	cytokine-cytokine receptor interaction	CCL13, CX3CR1, CCR1, CXCL14, CXCL12, CCL5, IL1B, OSM, CCR2, CXCL1, CCL18, TNF	AL, HF, HPT, OB
hsa04623	cytokine DNA-sensing pathway	CCL5, IL1B, CASP1	HF, HPT, SLS
hsa03320	PPAR signaling pathway	FABP5, SORBS1, LPL	HC, SLS, DB
hsa04064	NF-kappa B signaling pathway	CD14, BCL2A1, IL1B, TNF, LYN, CD14	DB, HPT, ST

### 3.2 Evaluating GO and KEGG pathways using enrichment analysis

We assessed eleven risk factors associated with ATH and examined their enrichment in GO and KEGG pathways. Specifically, we focused on the top five GO terms for each category: biological process (BP), cellular component (CC), and molecular function (MF), as well as five KEGG pathways. The top 5 highly enriched biological processes included the negative regulation of intracellular transport, negative regulation of leukocyte degranulation, positive regulation of cytokine production, positive regulation of interleukin-8 production, positive regulation of interleukin-8 secretion. In terms of cellular components, examples included clathrin-coated endocytic vesicles, the clathrin-coated endocytic vesicle membrane, integral plasma membrane, integral components of luminal side of endoplasmic reticulum membrane, and lysosome. Molecular functions that stood out included chemokine binding, phosphatidylinositol phospholipase C activity, chemokine receptor activity, and MHC class II receptor activity, phospholipase activity. The enriched KEGG pathways encompassed the chemokine signaling pathway, cytokine-cytokine receptor interaction, cytokine DNA-sensing pathway, PPAR signaling pathway and NF-kappa B signaling pathway.

### 3.3 Identification of regulatory biomolecules

**Fig. 3:**
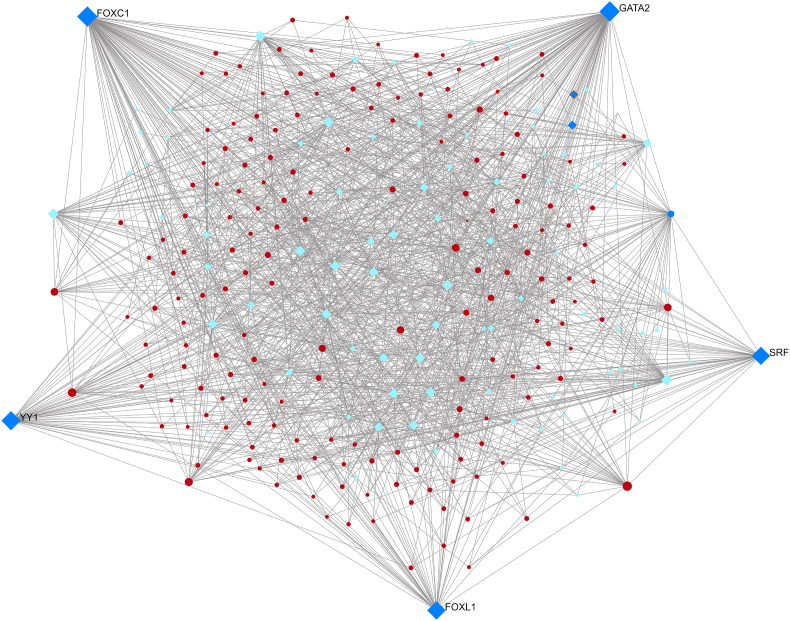
The interaction of differentially expressed genes and transcription factors were analysed to identify the transcription factors that control differentially expressed genes in ATH. Square shaped (blue and cyan color) indicates transcription factor, round shaped (red color) indicates differentially expressed genes. (For interpretation of the references to color in this figure legend, the reader is referred to the Web version of this article.)

**Fig. 4:**
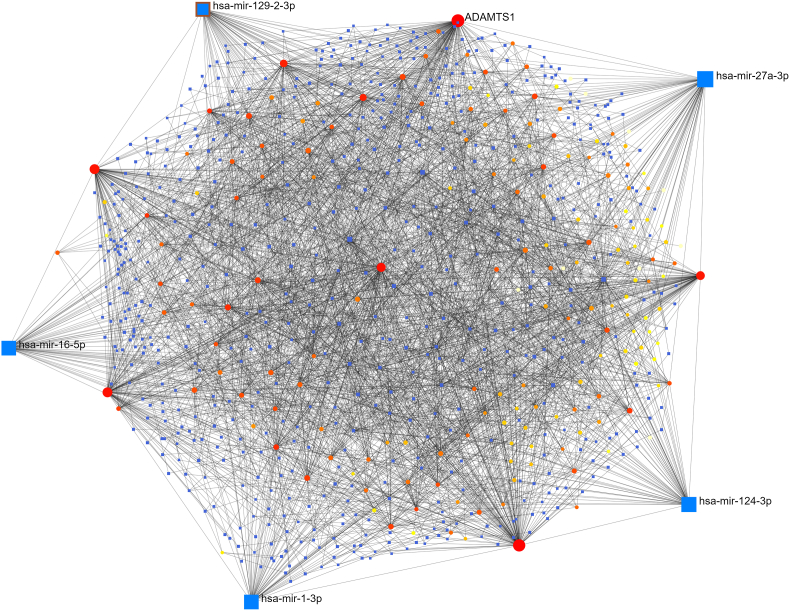
The interaction of differentially expressed genes and microRNAs were analysed to identify the microRNAs that control differentially expressed genes in ATH. Square shaped nodes (blue color) indicate microRNA and, circular shaped nodes (red and yellow color) indicate differentially expressed genes. (For interpretation of the references to color in this figure legend, the reader is referred to the Web version of this article.)

We investigated the common DEGs regulators using DEGs-TFs and DEGs-miRNAs interaction research, as shown in Table 2. By analysing topological parameters, we were able to identify interactions between DEG-TFs (Fig. 3) DEG-miRNAs (Fig. 4) and centrally regulating biomolecule from the interaction networks between DEGs-TFs and DEGs-miRNAs, five TFs (YY1, FOXL1, FOXC1, SRF, GATA2) and five miRNAs (mir-27a-3p, mir-124–3p, mir-16–5p, mir-129-2-3p, mir-1-3p) were identified respectively. These biomolecules regulate gene activity at both transcriptional and post transcriptional levels. YY1 TF raises the prevalence of cardiac failure in the general population [44]. FOXL1 TF is associated with pancreatic ductal adenocarcinoma in humans [45]. FOXC1 TF renders individuals more susceptible to cardiac failure [46]. SRF TF promotes cell division and proliferation [47]. The transcription factor GATA2 is linked to early-onset familial coronary artery disease [48]. Single strand RNA molecules are known as micro-RNA. They are small in size and typically have an average number of 22 nucleotides. They control post-transcriptional gene expression. We have identified several miRNAs.

**Table 2:** Overview of transcriptional and post-transcriptional regulators (TFs and miRNAs) of deferentially expressed genes.

	Symbol	Description	Feature
TF	YY1	YY1 TF	TF increase higher rates of cardiac failure [54]
	FOXL1	Forkhead Box L1	linked with human pancreatic ductal adenocarcinoma [55]
	FOXC1	Forkhead Box C1	play critical role in early cardio myogenesis [56]
	SRF	serum response factor	stimulates both cell proliferation and differentiation [57]
	GATA2	GATA Binding Protein 2	related with early onset familial coronary artery disease [58]
miRNA	mir-27a-3p	MicroRNA 27	involved in atherosclerosis formation by angiogenesis, apoptosis, lipid regulation and cytokine production [59]
	mir-124–3p	MicroRNA 124	reduces the production of collagen in atherosclerotic plaques [60]
	mir-16–5p	MicroRNA 16	mainly responsible for the coronary artery disease [61]
	mir-129-2-3p	MicroRNA 129	elevated level production may cause stroke [62]
	mir-1-3p	MicroRNA 1	plays a significant role on the control of cardiomyocyte apoptosis [63]

We have identified several miRNAs responsible responsible for regulating the DEGs. Here, we focus on five of these miRNAs: mir-27a-3p, primarily located in the endothelium, plays a role in processes such as angiogenesis, apoptosis, lipid regulation, and cytokine production. These functions collectively contribute to the development of atherosclerosis [49], mir-124–3p inhibits the production of collagen in atherosclerotic plaques [50], mir-16–5p is mainly responsible for the coronary artery disease [51], the elevated level of mir-129-2-3p may be a cause of stroke [52] and mir-1-3p plays a significant role in the regulation of cardiomyocyte apoptosis [53].

### 3.4 Analysis of PPI network

**Fig. 5:**
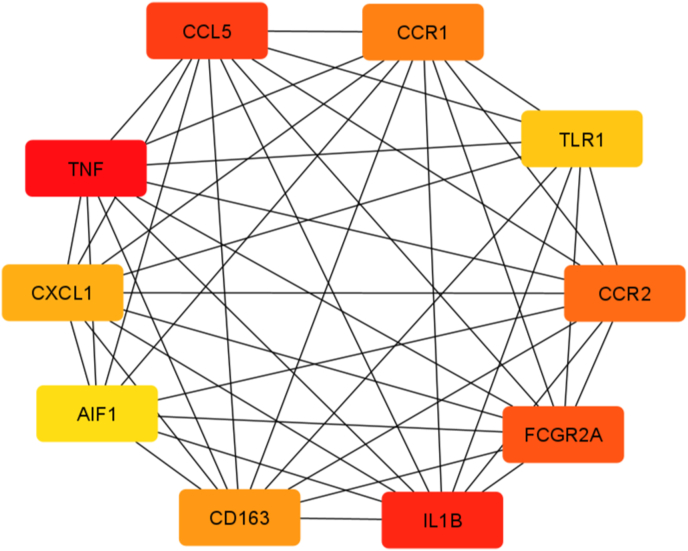
Simplified PPI network of differentially expressed genes shared by ATH and other risk factors is shown in the following figure. It highlights ten important hub proteins. Red, orange, and yellow colors indicate high, moderate, and low degrees of association, respectively. Darker colors correspond to higher degree of association. (For interpretation of the references to color in this figure legend, the reader is referred to the Web version of this article.)

**Fig. 6:**
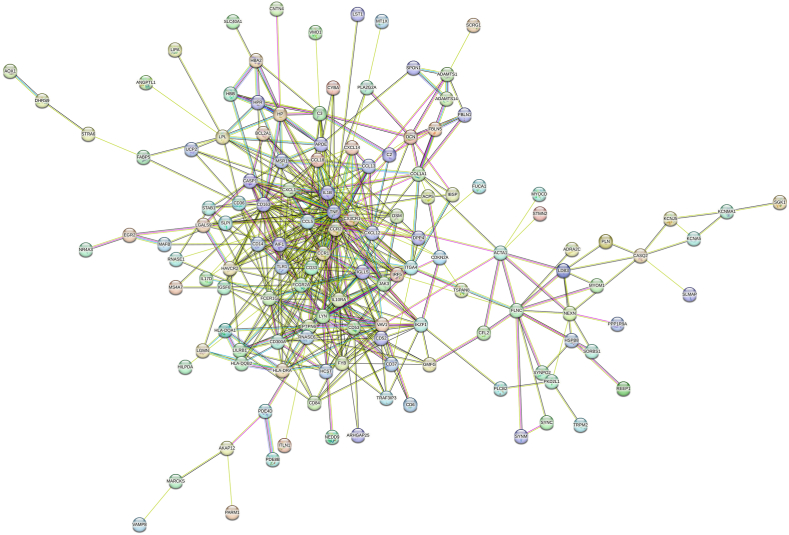
PPI network of differentially expressed genes shared by ATH and other risk factors.

The PPI network was developed by combining 225 unique DEGs shared by the ATH and its risk factors (Fig. 5, Fig. 6). In order to identify protein clusters with a high degree of connectivity, the topological analysis was applied using degree matrices. In the network, each node stands for a protein, and each terminal represents a protein-protein interaction. PPI analysis revealed ten hub proteins including CCL5, CCR1, TLR1, CCR2, FCGR2A, IL1B, CD163, AIF1, CXCL-1, and TNF. Perhaps this hub protein can used as therapeutic targets.

## 4 Discussion

The molecular networks associated with ATH and its risk factors were explored in our studies. We carried out a study on ATH gene expression data from peripheral arteries in carotid, femoral and infra-popliteal territories, comparing atherosclerotic and control tissue to recognize common DEGs between ATH and its risk factors. ATH tissues have been shown to be susceptible to 34 SM genes, 16 OB genes, 10 DB genes, 7 SLS genes, 11 HF genes, 82 AL genes, 37 ST genes, 5 HLP genes, 7 ADT genes, 9 HPT genes, and 9 HC genes. In order to determine the biological significance of the identified DEGs, GO and Molecular pathways analysis were performed, which revealed pathways with significantly altered activity. These pathways include the chemokine signaling pathway, which play a significant role in the inflammatory reactions connected to atherosclerosis [64]. The cytokine-cytokine receptor interaction is another important pathway that has a critical role in the development, progression, and complications of atherosclerosis [65]. Additionally, the activation of the inflammatory pathway by the cytoplasmic DNA-sensing pathway may aid in the development of ATH [66]. The Peroxisome Proliferator-Activated Receptor (PPAR) signaling pathway has also been implicated in inflammation-related ATH [67]. Moreover, nuclear factor kappa B (NF-kappa B) signaling pathway is also responsible for several types of inflammatory diseases related to ATH [68].

PPI analysis can provide some specific information about the primary mechanism of the disease. Therefore, we rebuilt the PPI networks by using the protein encoded by DEGs. Our topological study suggested ten hub proteins (CCL5, CCR1, TLR1, CCR2, FCGR2A, IL1B, CD163, AIF1, CXCL-1, and TNF) that are involved in ATH. A brief description of hub proteins list is given in Table 3. Among the hub proteins, CCL5 plays a role in immune regulation, inflammation, and is expressed on macrophages and T cells which are connected to ATH [69,70]. The hub protein CCR1 inhibits excessive plaque growth and inflammation [70], ATH plaque development was accelerated by the TLR1 protein [71], Leukocytes can enrol atherosclerotic vessels with the aid of the hub protein CCR2 [70]. IL1B is responsible for both acute and chronic inflammation [72], and FCGR2A increases susceptibility to peripheral atherosclerosis [73]. AIF1 supports macrophage in forming an ATH plaque [74]. CXCL-1 protein has an up-regulating function in ATH [75], and TNF increases the potential for CVD [76]. CD163 protein is expressed on macrophages, and elevated levels indicate ATH [77].

**Table 3:** List of ten hub proteins from the PPI network.

Symbol	Description	Gene Ontology	Feature	Risk Factors
CCL5	C–C motif chemokine ligand 5	chemokine receptor binding	involved in immunoregulatory and inflammatory processes	HF
CCR1	C–C motif chemokine receptor 1	enables C–C chemokine binding	alters the immuno-inflammatory activity in atherosclerosis and reduces excessive plaque development and inflammation	HPT
TLR1	toll like receptor 1	enables NAD(P) + nucleosidase activity	enhanced in human atherosclerotic plaques	SLS
CCR2	C–C motif chemokine receptor 2	enables CCR2 chemokine receptor binding	play key roles in leukocyte recruitment into the atherosclerotic vessels	HF, OB
FCGR2A	Fc gamma receptor II a	enables protein binding	increased susceptibility to peripheral atherosclerosis	AL
IL1B	Interleukin 1 beta	enables cytokine activity	increase the buildup of inflammatory cells in blood vessels and their invasion into the local intima of blood vessels	HPT
CD163	CD163 molecule	enables protein binding	functions primarily as a hemoglobin (Hb) scavenger receptor	OB
AIF1	allograft inflammatory factor 1	enables actin filament binding	mainly including allograft rejection, autoimmune disease, central nervous system (CNS) injury, vasculopathy and cancer	SM
CXCL-1	C-X-C motif chemokine ligand 1	enables CXCR chemokine receptor binding	plays a central role in macrophage accumulation and lesion progression	HPT
TNF	tumor necrosis factor	enables cytokine activity	regulates leukocytes activation, maturation, cytokine and chemokine release and generation of reactive oxygen and nitrogen	HPT

## 5 Conclusion

The genetic association of ATH with various diseases was discovered in this study through comprehensive transcriptomics analyses with human biomolecular networks. In order to identify potential key signaling and regulatory biomolecules in ATH, we identified candidate biomarkers at the RNA (transcripts and miRNAs) and protein levels (hub proteins). Possible molecular pathways involving ATH were also identified. This study provides new gene-based recommendations for disease diagnosis, and the molecular signatures of this biomarkers presented in this study may be value able for developing new treatments for ATH and conduction additional experiments studies on ATH.

## Funding

No funding was received.

## Author contributions

Conceptualization: Hossain Mohammad Hridoy, Md. Abdul Aziz, Md. Tofazzal Hossain.

Data curation: Hossain Mohammad Hridoy, Arnob Sarker.

Formal analysis: Hossain Mohammad Hridoy, Md. Nasim Haidar.

Methodology: Hossain Mohammad Hridoy, Md. Tofazzal Hossain, Md. Pervez Hossain.

Writing (Drafting): Chadni Khatun, Hossain Mohammad Hridoy.

Writing (Editing & Review): Md. Tofazzal Hossain, Md. Abdul Aziz.

Supervision & Project Administration: Md. Tofazzal Hossain.

Validation & Visualization: Md. Tofazzal Hossain, Md. Abdul Aziz.

## Declaration of competing interest

The authors declare that they have no known competing financial interests or personal relationships that could have appeared to influence the work reported in this paper.
